# Acceptance rate of clinical pharmacists’ recommendations—an ongoing journey for integration

**DOI:** 10.3389/fphar.2023.1253990

**Published:** 2023-09-13

**Authors:** Orit Peled, Yael Vitzrabin, Eran Beit Ner, Moran Lazaryan, Maya Berlin, Dana Barchel, Matitiahu Berkovitch, Yiftah Beer, Eran Tamir

**Affiliations:** ^1^ Department of Pharmacy, Schneider Children’s Medical Center of Israel, Affiliated to School of Medicine, Tel Aviv University, Petach Tikva, Israel; ^2^ Pharmacy Department, Yitzhak Shamir Medical Center, Zerifin, Affiliated to School of Medicine, Tel Aviv University, Tel Aviv, Israel; ^3^ Department of Orthopaedic Surgery, Yitzhak Shamir Medical Center, Zerifin, Affiliated to School of Medicine, Tel Aviv University, Tel Aviv, Israel; ^4^ Clinical Pharmacology and Toxicology Unit, Shamir Medical Center, Zerifin, The Andy Lebach Chair of Clinical Pharmacology and Toxicology, Tel-Aviv University, Tel-Aviv, Israel; ^5^ Diabetic Foot Unit, Yitzhak Shamir Medical Center, Zerifin, Affiliated to School of Medicine, Tel Aviv University, Tel Aviv, Israel

**Keywords:** pharmacology, clinical pharmacist, integration, acceptance, drug-related problem, challenge, multidisciplinary

## Abstract

**Introduction:** Multidisciplinary expert team collaboration in the clinical setting, which includes clinical pharmacist involvement can facilitate significant improvements in outcomes and optimize patient management by preventing drug-related problems (DRP). This type of collaboration is particularly valuable in patients with multi-morbidity and polypharmacy such as diabetic foot patients. Evidence regarding the successful integration of a new clinical pharmacist, without previous experience into a unit is still scarce. Therefore, this study aimed to describe and evaluate the actual successful integration process of the clinical pharmacist into a diabetic foot unit by measuring the change in recommendation acceptance rate over time.

**Methods:** A prospective, exploratory treatment effectiveness study based on the recommendation acceptance rate of a new clinical pharmacist introduced into the diabetic foot unit was conducted over a 9- month period. The clinical pharmacist identified medical and drug-related problems (DRP) or any discrepancies in the prescribing and administration of medications. Each identified DRP was documented and formulated as a recommendation by the clinical pharmacist. The main outcome measure was the acceptance rate of recommendations over time.

**Results:** A total of 86 patients, of which 67% were men, averagely aged 66.5 (SD 11.8) years were evaluated. Calculated BMI was 30.2 (SD 6.2). The average number of medical diagnoses was 8.9 (SD3.2), and 11.1 (SD 3.7) prescribed drugs for each patient. Cardiovascular disease was presented by 95% (*n* = 82) of the patients and 33% of them (*n* = 28) had uncontrolled hyperglycemia. Averagely, 3.3 (SD 1.9) DRPs were identified pre patient. The efficacy-related DRP recommendation acceptance rate increased over the study period from 37.8% in the first 4 months to 79.4% after a period of 4.75 months. Safety-related DRP recommendation acceptance rate increased from 56% to 67.6%.

**Conclusion:** Improved clinical outcomes and optimized pharmacologic patient management may be achieved by the successful integration of a clinical pharmacist into the team. This study provides evidence of the increasing recommendation acceptance rate of integrated, pharmacist-driven comprehensive medication management in an unexperienced unit. To overcome challenges, team members should collaborate to fully integrate the clinical pharmacist into the team-based structure and utilize proper strategies to minimize and transcend barriers.

## Introduction

In today’s practice environment, which includes increasingly complex medication management and increased performance expectations, collaborating with clinical pharmacists is a promising way to improve and expand team-based care. Clinical pharmacists possess the education, clinical training expertise and experience to provide substantial value to patient care by minimizing potential drug-related issues and optimizing drug therapy (Nichols-English et al., 2002; Pousinho et al., 2016; Korcegez et al., 2017). While it was not until the early 1960s that the healthcare model adapted to allow pharmacists a more substantial clinical role, (Pearson, 2007; LLC AH, 2014), by the early 2000s, the scope of practice for pharmacists within patient care further expanded despite challenges (Pearson, 2007). Today, the role of clinical pharmacists is still evolving with wide regional variations in pharmacist roles, regulations, and educational qualifications. In countries like the United States, United Kingdom and Australia, the establishment of Pharm D programs and independent prescribing regulations have significantly advanced the role of pharmacists in clinical services (Abousheishaa et al., 2020; Ahmer Raza et al., 2022). However, in other countries, the impact of clinical pharmacists on patient care is still evolving and adapting to current gaps in the healthcare system (Said et al., 2022). In Europe and the United Kingdom, the scope of clinical pharmacy practice is on pharmacy inspection, medication management, and ensuring quality of care, with generally more limited ability for clinical pharmacists to prescribe compared to the United States (Abousheishaa et al., 2020; Ahmer Raza et al., 2022). In other nations like Israel, clinical pharmacists primarily focus on medication counseling, guideline development, and medication-related research (Rose et al., 2021; Schwartzberg and Marom, 2021). While advanced-degree pharmacists are theoretically qualified to prescribe medications, limited authority and practical barriers like infrastructure limitations and reimbursement issues have made this difficult to implement (Rose et al., 2021; Schwartzberg and Marom, 2021). Consequently, in many countries like Europe and Israel, although there are provisions suggesting that pharmacists with MSc or PharmD degrees should be able to prescribe, for now, practical regulations and complexities hinder this evolution. It has been widely investigated and demonstrated that pharmacist–physician collaboration significantly improves clinical outcomes (Willi et al., 2008; Kelly et al., 2013; Hwang et al., 2017; Matzke et al., 2018; Vinterflod et al., 2018; Albassam et al., 2020). However, to achieve true optimization of pharmaceutical treatment, pharmacist intervention might be insufficient, especially in counties without regulatory frameworks for pharmacist practice. Acceptance of the pharmacist’s recommendations by the prescribers and supporting staff is a crucial step in the process of amending and improving patient management. Conversely, unsuccessful implementation of clinical pharmacists’ recommendations would lessen their contribution, and likely compromise patients’ health (DeName et al., 2008; Wong, 2017).

While initially challenging, the integration of a clinical pharmacist offers substantial potential benefits. There are a numerous studies in the medical literature discussing the barriers and provide recommendations to assist pharmacists to successfully integrate into existing primary care teams (Galt et al., 1999; Jorgenson et al., 2013; Jorgenson et al., 2014; Hayhoe et al., 2019; Kempen et al., 2020; Said et al., 2022). To overcome such challenges, team members must work together to implement the team-based structure (Jorgenson et al., 2013). Unfortunately, recent evidence suggests that pharmacists often continue to make the same mistakes and struggle to integrate into these teams, despite the fact that these barriers are well documented in the literature (Jorgenson et al., 2014). There remains, however, a lack of comprehensive evaluated descriptions emphasizing the integration process itself over time, measured as compliance outcomes achieved by overcoming the challenges.

Patients with several medical conditions and polypharmacy have complex health needs. Diabetes Mellitus is an example of a chronic disease that requires proper medical care along with the education of healthcare providers and patients to prevent its short- and long-term complications (American Diabetes Association, 2003). After physicians and nurses, pharmacists are the third largest group of healthcare providers in the world who can provide patient education (Chan and Wuliji, 2023).

Foot ulcer, a common complication among diabetic patients, is a leading cause for emergency department visits and hospital admissions and may cause severe life-threatening sepsis and amputations. Management of diabetic foot patients is extremely challenging due to poor treatment effectiveness and low compliance for both local foot treatments and other diabetic comorbidities. The pharmacologic aspect of the complexity of patients suffering from diabetic foot disease results from multiple drug consumption, and leads to increased risk of drug-related problems (DRP’s), including life-endangering drug interactions and administration errors (Shareef et al., 2016; Breuker et al., 2017). Optimized medical treatment in this population can improve clinical outcomes in these patients, reducing morbidity and mortality, as well as improving their quality of life (McLennan et al., 1999; Boulton et al., 2005; Shareef et al., 2016). Understanding this complexity, the American Diabetes Association recommends a multi-disciplinary approach composed of a professional expert team, including both clinical pharmacists and clinicians (Lipsky et al., 2012). Some counties, like United States, United Kingdom and Australia, advanced beyond these recommendations which have already significantly impacted clinical pharmacists engagement in clinical services and the extent of their involvement in patient care (Abousheishaa et al., 2020; Ahmer Raza et al., 2022). Nevertheless, in other nations, the role of pharmacists and their impact on patient care is still evolving and adapting to current gaps in the healthcare system (Rose et al., 2021; Schwartzberg and Marom, 2021). While the integration of pharmacists into multidisciplinary teams is increasingly common, the influence of pharmacist-led interventions on diabetic patient-related outcomes and diabetic foot complications has been assessed by few studies (Jameson and Baty, 2010; Lipsky et al., 2012; Ip et al., 2013; Cahn et al., 2014; Pousinho et al., 2016; Soprovich et al., 2019; Al-Taie et al., 2020; Alsuwayni and Alhossan, 2020; Khan et al., 2021; Rebolledo et al., 2022).

This study aims to evaluate the acceptance rate of a clinical pharmacist’s recommendations by the healthcare providers of a unit who had yet to collaborate with a clinical pharmacist and without regulatory frameworks for pharmacist practice. This evaluation will focus on the actual integration process and the evolving compliance outcome of recommendation acceptance rate over time with detailed steps taken to address the barriers. This way, the study can provide a more in-depth understanding of how successful integration is achieved in applicable medical centers and its impact on patient care outcomes.

## Methods

### Study design and professionals

A prospective, interventional study was conducted over 9 months in 2019 at the diabetic foot unit of “Shamir” (Assaf Harofeh) Medical Center in Israel. During this period, a new clinical pharmacist who lacked any previous experience as an in-ward clinical pharmacist was introduced to the unit as part of the multidisciplinary medical team. A clinical pharmacist is a specialized healthcare professional who works directly with patients and healthcare teams to ensure safe and effective medication use, optimize drug therapy, and improve patient outcomes. The clinical pharmacist in this study completed a four-year bachelor’s degree program in pharmacy, followed by a two-year master’s degree in “Clinical Community Pharmacy and Regulatory Management.” Throughout studies, extensive experience as a clinical pharmacist was gained, developing skills in supervision, pharmacist counselling, policy development, and optimization processes in community settings involving both public and private sectors. The clinical pharmacist acquired additional experience in both a community pharmacy and the hospital’s Pharmacy Department. Although the program provided some required skills, an internship in an inpatient ward was not mandatory as it was not the program’s primary focus. Integrating into the diabetic foot unit served as first experience in an inpatient ward setting. The study involved screening patient records, identifying drug-related problems (DRPs), evaluating patient treatments, and formulating recommendations, which were integral components of the studies and training.

The clinical pharmacist was present in the ward 40%–60% (16–24 per week in 5 days) of a full-time position in the hospital. The study was approved by the Ethics Committee of Shamir Medical Center.

### Study population

Diabetic foot infection patients over the age of 18 years old who were admitted to the diabetic foot unit were included in this study. Pregnant women and patients with active malignancy were excluded. As the pharmacist’s aim was to provide the greatest impact to the patients’ ward, patients that will most likely present DRPs and benefit the most from the intervention were selected. Those patients are the most complicated patients, presenting multiple comorbidities, polypharmacy or medications with high-risk factors for DRP. Additionally, patients could also be referred to the clinical pharmacist by the medical team if a comprehensive medication management was needed.

### Data extraction

A comprehensive review of the patient’s medical history and records was conducted as part of routine management by the clinical pharmacist. In addition to screening patient’s records, individualized patient encounters were included when needed with the patient or family relative conducted in a face-to-face manner.

Patient demographic characteristics, and medical conditions from the past and during the respective hospitalization were recorded as were laboratory test results. Prescribed medications and dietary supplements were documented, including information regarding the formulation, dosage, and administration regimen.

In addition, the pharmacist recorded collateral information related to admission and medication reconciliation. Based on the medication review, the clinical pharmacist assessed the patient’s medication therapy and evaluated its appropriateness, safety, and effectiveness in achieving treatment goals.

### Variables

Patients’ records were screened by the newly introduced clinical pharmacist in the unit in order to detect drug-related problems (DRP) and to formulates evidence-based recommendations to address any identified DRP. DRPs were classified into one of eight categories according to Strand’s model (Strand et al., 1990) which evaluates pharmacologic patient management: 1) untreated indication; 2) improper drug selection; 3) sub-therapeutic dosage; 4) failure to receive drugs; 5) overdose; 6) adverse drug reactions; 7) drug interactions and 8) drug use without indication. In addition, researchers recorded whether DRPs were broadly related to medication safety or efficacy. Although the issues of medication safety and efficacy sometimes overlapped or concomitantly occurred within the same assessment, DRPs that were classified as “safety” related included assessments that could actually or potentially result in drug toxicity and related adverse effects.

For better comprehensive drug-related management and to cover the entire spectrum of drug-related information required for the pharmacist, two informative categories were added: missing information required and specialist consultation required.

Once the DRP’s were classified, the severity of each DRP was assessed according to a slightly modified version of Overhage et al., generating a categorical variable with five categories: 1) potentially fatal, 2) serious, 3) significant, 4) minor and 5) error free that was also used in similar studies (Overhage and Lukes, 1999; Fernández-Llamazare et al., 2013) The assessment of DRPs and severity ratings were performed by two pharmacists independently, and discrepancies were resolved through discussions.

### Intervention

Following a thorough review of each patient’s medical record, and once the DRPs were classified, the clinical pharmacist formulated evidence-based recommendations regarding medication management changes to address any DRP. Both DRP classification and pharmaceutical recommendations were made according to updated guidelines and literature, as well as computerized pharmacological databases and a clinical decision support platform which interfaces with the electronic medical records, identifying any potential DRPs (Shah et al., 2021).

The resolving recommendations made by the pharmacist were classified according to a modified taxonomy described by Hoth et al. as follows: 1) de-prescribe medication (renamed from original “discontinue medication”), 2) hold medication, 3) start or restart medication, 4) start alternative therapy, 5) change dose, 6) change route, 7) change time-of-day administered, 8) change dosage strength, 9) change dosage form, 10) change duration of treatment, 11) recommend patient or prescriber education, and 12) recommend laboratory or symptom monitoring (Hoth et al., 2007). Both the resolving recommendations made by the clinical pharmacist and their classifications were supervised by a senior well experienced clinical pharmacist engaged to review and discuss the different interventions, allowing better reliable interventions and clinical outcomes, as well as enhance better experience to the new clinical pharmacist.

The clinical pharmacist communicated the recommendations to the healthcare team, which may include individual physicians, medical residents, nursing staff, and other healthcare professionals involved in the patient’s care. During the independent process of review and classification, sessions with the medical staff were held to provide the recommendations and to assess their responsiveness to the given recommendations. These sessions included daily participation in morning rounds which included multidisciplinary medical team. During rounds, recommendations may be provided for multiple patients, allowing for real-time discussions and immediate implementation of changes. Additionally, participating in relevant group discussions or multidisciplinary team meetings were used to present recommendations for multiple patients in a structured manner. In specific patients, communicating directly to individual physicians through one-on-one interactions could be conducted in the cases of urgent intervention or just using electronic health record systems when more appropriate. Any recommendation was recorded in the patient’s record system. Therefore, any healthcare professional, before or after shifts exchanges, in any timing, could be exposed to the information. In this way correct follow-up information is communicated between the different healthcare professional, and continuity of the pharmacist’s activities can be kept without meeting in person.

In-depth discussions and opinion exchange increased awareness and improved compliance of the medical staff to the clinical pharmacist’s recommendations and therefore integration.

### Outcomes

The final decision was made by the respective medical healthcare provider to assess the compliance and agreement of the medical staff with the pharmacist’s recommendations (i.e., accepted, rejected). The rate of the recommendations’ acceptance in accordance with the DRP classification, severity rating, and recommendation classification was the main outcome measurement of this study. The secondary outcome was the change in acceptance rate over time during the integration period of the clinical pharmacist in the unit.

### Data analysis

Data were presented using descriptive statistics, with means, ranges, and standard deviations for continuous variables, frequencies, and percentages for dichotomous variables.

The rate of recommendation acceptance was analyzed and presented as an average of acceptance rate in a moving time window of 120 days, measured every 3 days. An alpha level of 0.05 was set to determine statistical significance. Analyses were performed using Microsoft Excel (Microsoft 2013; Redmond, WA).

## Results

### Patients characteristics

A total of 86 patients were screened during their hospitalization by a newly incorporated clinical pharmacist in the Diabetic Foot unit for a period of 9 months in 2019. Population demographics and characteristics are depicted in Table 1. Sixty-seven percent of the study group were men. The average population age was 66.5 (SD 11.8) years with the majority being over the age of 50 years (89.5%). The average BMI was 30.2 (SD 6.2) distributed to 26% defined as “overweight” (BMI ≥25), 30% of them “obese” (BMI ≥30) and 20% “extreme obese” (BMI ≥35).

**TABLE 1:** Demographic and clinical characteristics of the study participants (*n* = 86).

Variable	Value
Age (year), mean ± SD	66.5 ± 11.8
Range (year)	36–93
Gender, *n* (%)	
Male	58 (67%)
Female	28 (33%)
Cigarette smoking (*n* = 83), *n* (%)	
Yes	16 (19%)
Former smoker	16 (19%)
No	51 (61%)
BMI, mean ± SD	30.2 (6.2)
BMI, *n* (%)	
<25	21 (24%)
25–30	22 (26%)
30–35	26 (30%)
35–40	12 (14%)
>40	5 (6%)
DM disease duration (year) ± SD	19.5 (8.7)
Medical Diagnosis/patient ±SD	8.9 (3.2)
Number of medications/patients ±SD	11.1 (3.7)
Number of DRP’s/patient ±SD	3.3 (1.9)
Medical conditions, n (%)	
DM without hypertension	9 (10%)
DM plus hypertension and hyperlipidemia	66 (77%)
DM plus hypertension and other	11 (13%)
DM medication regimen type, n (%)	
Insulin based	59 (69%)
Non-insulin based	27 (31%)

Abbreviations: SD, standard deviation; BMI, body mass index; DM, diabetes mellitus.

The average number of medical diagnoses recorded for each patient was 8.9 (SD 3.2) with an average number of prescribed drugs of 11.1 (SD 3.7). Cardiovascular disease was presented by 95% (*n* = 82) of the patients and 33% of them (*n* = 28) had an uncontrolled glucose level, and were advised to improve their glycemic state.

### DRP assessment and acceptance rate

A total of 286 DRPs were identified in the 86 screened records, an average of 3.3 (SD 1.9) DRPs for each patient. Two patients had eight DRPs while only two patients did not have any. No correlation was identified between the number of prescribed medications and the number of DRPs, r (84) = 0.10, *p* = 0.35.

**FIGURE 1:**
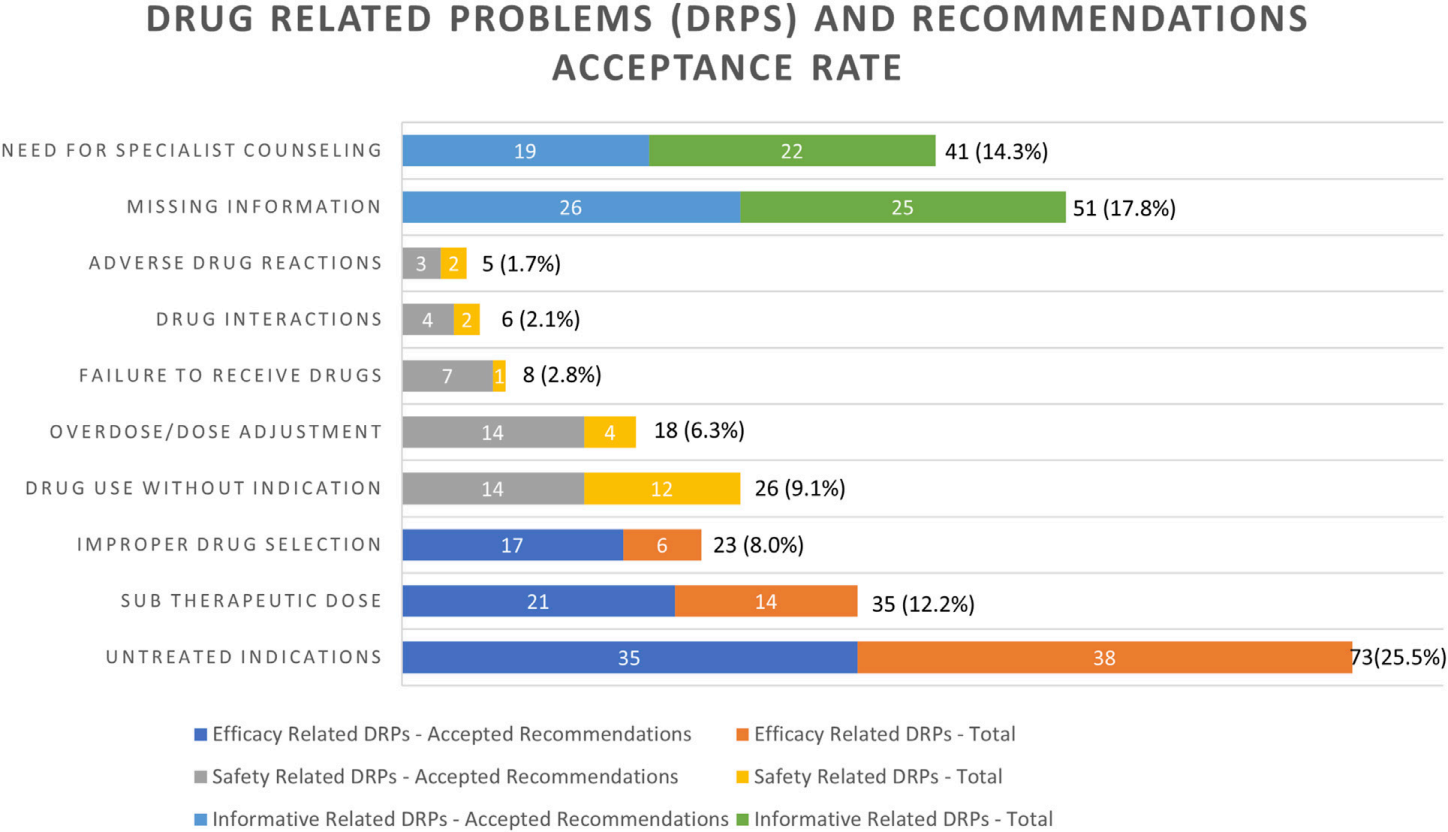
Frequencies of drug-related problems (DRPs) identified and recommendations issued by the clinical pharmacist’s acceptance rate. Variables are expressed as number and percentage of the total (*n* = 286) DRPs, *n* (%).

Figure 1 presents the assessment of different DRPs identified by the clinical pharmacist and their respective acceptance rate. The most frequently documented DRP, an untreated indication, was reported in 25.5% of the cases (*n* = 73). Missing information was identified in 17.8% (*n* = 51), of these, most of the events were the result of missing laboratory tests (90.6%) and the response was a recommendation for completion of the required test. Need for specialist consultation was the third most frequently reported DRP in 14.3% (*n* = 41) of cases followed by sub-therapeutic dose DRPs, which accounted for 12.2% (*n* = 35) of all DRPs. For these patients, a modification in drug dosage was advised.

Drug use without indication was identified in 26 (9.1%) cases. Eight percent (*n* = 23) of the DRPs were the result of an improper drug selection and 6.3% percent of the DRPs were associated with overdose (*n* = 18). Failure to receive drugs was identified in 2.8% (*n* = 8) of the incidents. Drug interactions and adverse drug reactions were responsible for 2.1% and 1.7% of the DRPs, respectively.

The two informative categories “missing information required” and “specialist consultation required” were responsible for 32.2% of all identified DRPs, with an acceptance rate of only 15.7% of the respective recommendations, while Strand’s model eight DRPs categories were responsible for 67.8% of all identified DRPs, with an acceptance rate of 40.2% of the respective recommendations (Strand et al., 1990) (Figure 1). Furthermore, 22.0% (*n* = 63) of DRPs identified were broadly related to medication safety concerns, whereas less were related to medication efficacy (*n* = 131, 45.8%). The acceptance rate of the recommendation of the medication safety concern-related DRPs was higher than the efficacy-related DRPs (14.7% vs. 25.5%, Figure 1). All the mentioned rates are respective to the total number of DRPs (286).

The most prevalent medications on any DRP were statins (*n* = 32, 14.3%), and the most prevalent medication-specific DRP that could be prevented was untreated indications (*n* = 68, 30.4%) (Table 3).

### Severity assessment and acceptance rate

**FIGURE 2:**
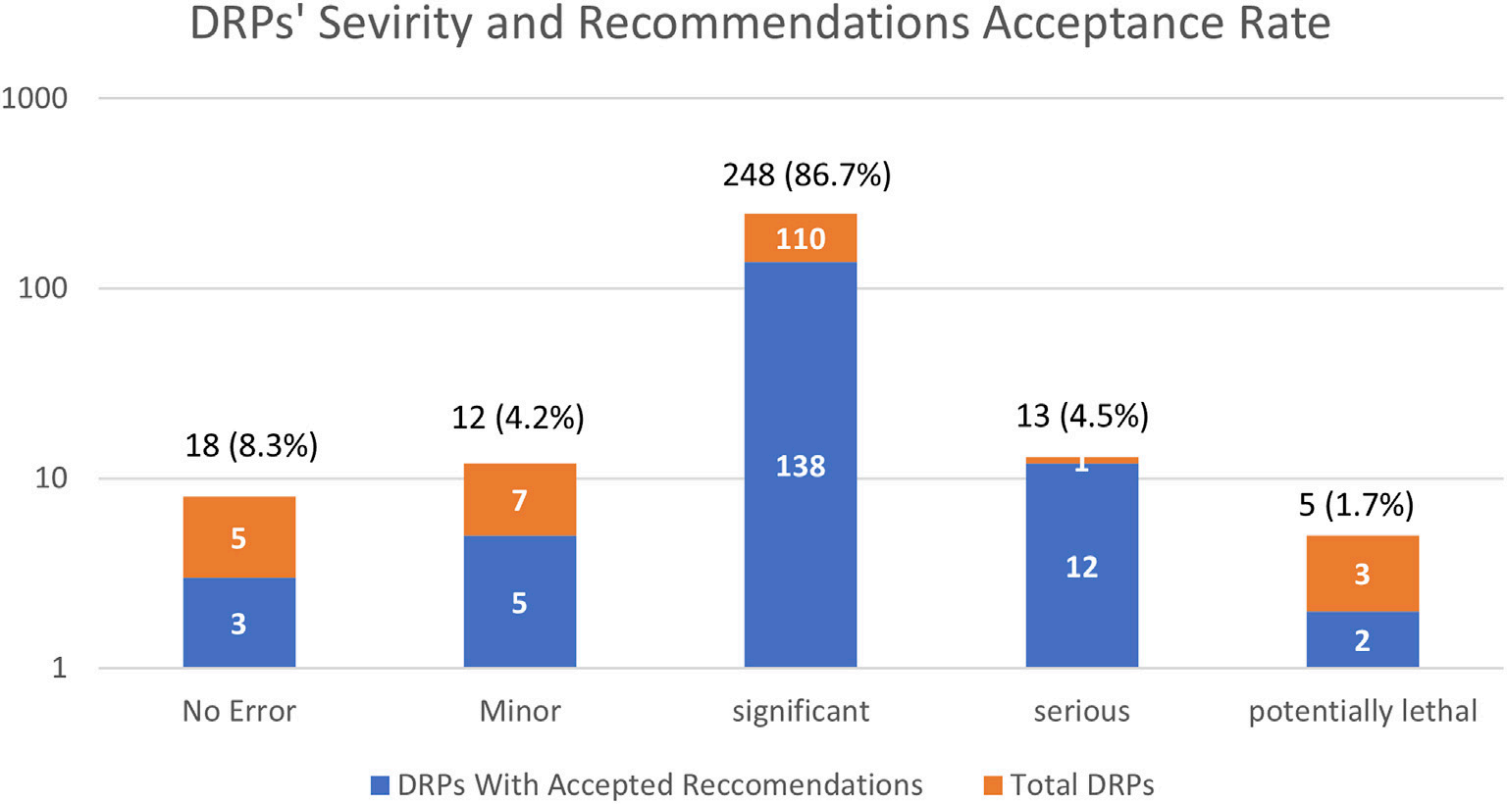
Frequencies severity of drug-related problems (DRPs) identified and recommendations issued by the clinical pharmacist’s acceptance rate presented in a logarithmic scale. Variables are expressed as number and percentage of total (*n* = 286) DRPs, *n* (%).

With regard to the clinical severity of the identified DRPs, most of them (86.7%, *n* = 248), were considered significant, 4.5% (13 cases) were clinically serious, 4.2% (12 cases) were of minor significance and 1.7% (5 cases) were potentially lethal (Figure 2). Both “serious” and “potentially lethal” severity levels addressed cases that were life-threatening. The acceptance rate of the respective recommendation of the DRPs increased according to the significance of the clinical severity (excluding the potentially lethal DRPs) as demonstrated by Figure 2.

### Recommendations and acceptance over time

Every identified DRP was answered with a clinical pharmacist’s advice. Table 2 details the types of recommendations the pharmacists made to resolve DRPs. The majority of DRPs categories matched to one main category of recommendation.

**TABLE 2:** Frequencies of drug related problems (DRPs) identified by the clinical pharmacist.

Recommend DRP	Addition of drug	Change in drug dose	Need for lab investigation	Cessation of drug	Need for patient counselling	Substitution of drug	Change in frequency of administration	Change in rout of administration	Change in dosage form	Sum
Need for specialist counseling					41					41 (14.3%)
Missing information	2		49							51 (17.8%)
Adverse drug reactions		2		3						5 (1.7%)
Drug interactions				4			2			6 (2.1%)
Failure to receive drugs	8									8 (2.8%)
Overdose/Dose adjustment		16		2						18 (6.3%)
Drug use without indication				26						26 (9.1%)
Improper drug selection	1	1		9		8	2	1	1	23 (8.0%)
Sub therapeutic dose		35								35 (12.2%)
Untreated indications	72	1								73 (25.5%)
Sum	83 (29.0%)	55 (19.2%)	49 (17.1%)	44 (15.4%)	41 (14.3%)	8 (2.8%)	4 (1.4%)	1 (0.3%)	1 (0.3%)	286

The most prevalent medications related to any recommendation were statins (*n* = 32, 14.3%), being also the medications with the highest recommendation acceptance rate (*n* = 20, 15.5%) (Table 3). The most prevalent medication-specific recommendation made by the clinical pharmacists to prevent DRPs was addition of drug (*n* = 78, 34.8%) being also the most prevalent medication-specific recommendation that was accepted by the physicians (*n* = 41, 31.8%) (Table 3).

**TABLE 3:** Most prevalent medications on DRP or recommendation and medication-specific information according to the different variables and outcome.

Most prevalent medications	Frequency (%)
Most prevalent on DRPs and recommendations^a^	224 (100%)
Statin	32 (14.3%)
Insulin	30 (13.4%)
Aspirin	20 (8.9%)
Proton Pump Inhibitor	16 (7.1%)
Clopidogrel	15 (6.7%)
Metformin	9 (4.0%)
Most prevalent on accepted recommendations	129 (100%)
Statin	20 (15.5%)
Insulin	17 (13.2%)
Aspirin	10 (7.8%)
Proton Pump Inhibitor	8 (6.2%)
Hydrochlorothiazide	6 (4.7%)
Clopidogrel	5 (3.9%)
Most Prevalent Medication-specific information	
Medication-specific DRPs	224 (100%)
Untreated indications	68 (30.4%)
Sub therapeutic dose	35 (15.6%)
Drug use without indication	27 (12.1%)
Medication-specific recommendations	224 (100%)
Addition of drug	78 (34.8%)
Change in drug dose	55 (24.6%)
Cessation of drug	44 (19.6%)
Medication-specific accepted recommendations	129 (100%)
Addition of drug	41 (31.8%)
Change in drug dose	37 (28.7%)
Cessation of drug	28 (21.7%)

^a^ Each medication with identified DRP, had a compatible formulated recommendation.

**FIGURE 3:**
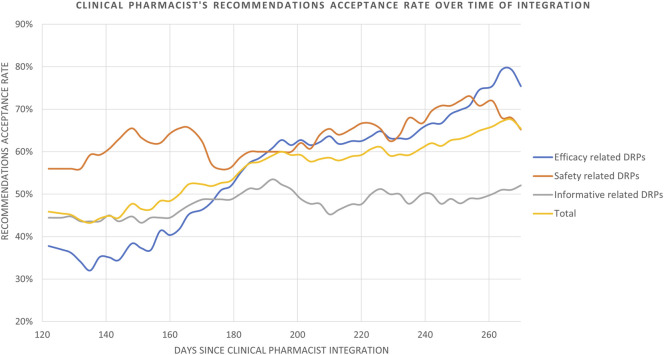
Clinical pharmacist’s recommendations acceptance rate over time of integration. Data is analyzed and presented as an average of acceptance rate in a moving time window of 120 days measured every 3 days.

The rate of recommendation acceptance increased over time from the clinical pharmacist’s initial recommendations until the end of the 9-month study period in the Diabetic Foot unit (Figure 3). The pharmacists’ recommendations regarding medication safety-related DRPs were accepted in 56.0% of the cases at the beginning, and reached 67.6% by the end of the study period. In contrast, a significantly lower acceptance rate was noted among recommendations that were efficacy-related DRPs at the beginning of the integration process (37.8%), however, these increased significantly by the end of the study period (79.4%). Therefore, a greater improvement is evident (depicted in Figure 3) in the acceptance rate of efficacy related vs. safety-related DRP recommendations.

The category of recommendations in response to information related DRPs which was not part of Strand’s 8 category DRPs, demonstrated only a slight improvement in acceptance rate, from 44.4% at the beginning to 52.1% by the end of the study period. Overall, the clinical pharmacist’s recommendations (*n* = 160) were accepted at a rate of 45.9% at the beginning, improving to 67.6% by the end of the study period (Figure 3). The rate was calculated with a moving average of 120 days measured every 3 days, in order to smooth the curve and depict curve behavior throughout the study period.

## Discussion

While integrating a clinical pharmacist into existing primary care teams may present initial challenges, the potential benefits are substantial. Numerous studies in the medical literature have explored the barriers and provided recommendations to facilitate successful integration (Durand et al., 2022). However, despite the well-documented barriers, recent evidence indicates that pharmacists often encounter persistent difficulties in fully integrating into these teams, and these challenges seem to recur. There is a scarcity of data on the actual integration process of a new clinical pharmacist in a medical unit without previous experience or understanding of the potential benefits, as well as lack of data on the change of recommendation acceptance rate over time. To our knowledge, this is the only study focusing on the change over time in the measurement of compliance to recommendations outcome achieved by overcoming these hurdles during the integration evolving process. The successful integration of clinical pharmacists and team collaboration was demonstrated by an increase in acceptance rate of the pharmacist’s recommendations over the course of the study period.

Clinical pharmacists possess the expertise to optimize pharmacologic patient management and play a crucial role in overseeing and ensuring appropriate and safe drug usage (Nichols-English et al., 2002; American College of Clinical Pharmacy et al., 2012; Pousinho et al., 2016). They are of great benefit in cases of multimorbidity and polypharmacy, as observed in our study population. In some countries, such as the United States, United Kingdom, and Australia, there are advanced regulations in pharmacist roles to significantly impact the engagement of clinical pharmacists in clinical services and the extent of their involvement in patient care (Abousheishaa et al., 2020; Ahmer Raza et al., 2022). In other countries and health systems, like in Europe and Israel, these advances are still dynamically evolving adapting to current gaps towards independent prescribing regulations (Abousheishaa et al., 2020; Rose et al., 2021; Schwartzberg and Marom, 2021; Ahmer Raza et al., 2022). In many countries, clinical pharmacists frequently encounter barriers such as undefined roles, inadequate pharmacist support and training, and as a result, are often unclear about the expectations regarding their responsibilities among team members and patients (Assa-Eley and Kimberlin, 2005; Newton et al., 2007; Farrell et al., 2008; Dey et al., 2011; Kozminski et al., 2011; Berdine et al., 2012). Pharmacists are also typically unfamiliar with the roles of other team members during the initial period, (Dobson et al., 2006), creating confusion (Gulliver et al., 2002; Gurn and ey, 2009; Goldman et al., 2010) and lack of assertiveness and thus, they often rely on other team members for assistance (Brock and Doucette, 2004).

As the integration process can typically take several months, often nine to 12, for pharmacists to become efficient in their new roles, our study utilized a nine-month integration period, during which there was an impressive change in acceptance rate. This suggests that the aforementioned challenges and barriers could potentially be minimized or prevented with adequate preparation and time (Jorgenson et al., 2013).

In the present study, the Diabetic Foot unit represents an example of a multidisciplinary healthcare team unexperienced with the process of integrating a clinical pharmacist. Our study group’s demographic characteristics align with the current literature, presenting with multiple comorbidities which require polypharmacy (Khan et al., 2021; Al-Taie et al., 2020; Dinh and Veves, 2008). Optimized drug treatment in these patients, particularly those with diabetic foot complications, can improve clinical outcomes, while improving quality of life (McLennan et al., 1999; Boulton et al., 2005; Shareef et al., 2016).

This study included 86 patients screened over 9 months period. However, the clinical pharmacist was present in the ward only 40%–60% (16–24 per week in 5 days) of a full-time position in the hospital. This is one of the reasons for the ostensible low average number of patients. The small number of participants can also be attributed to the properties and characteristics distinguishing the diabetic foot population in an inpatient ward compared with a general inpatient ward. This challenging population is characterized by complex medical conditions with multiple comorbidities, diverse age range, varying disease severity, and the need for polypharmacy management in addition to educational needs, and risk factors that add to the complexity of care. Moreover, it is important to consider that the clinical pharmacist lacked experience initially but was supported by a senior experienced clinical pharmacist in an unfamiliar ward where the potential benefits of having an inward clinical pharmacist were not widely recognized. Consequently, there was a low number of screened patients at the start of the study, which gradually improved during the integration period as the ward’s motivation to collaborate increased. Additionally, the pharmacist targeted patients who would benefit the most from the intervention by selecting those with complex medical conditions, multiple comorbidities, polypharmacy, and medications with high-risk factors for drug-related problems (DRPs). Therefore, due to the mentioned reasons the low average number of patients can be misinterpreted as low-level activity of the clinical pharmacist, whereas it was influenced by various factors such as targeting complicated patients, the learning curve and the evolving integration process.

Hepler and Strand’s novel article defined DRPs as a drug treatment event or circumstance that actually or potentially interferes with optimal patient outcome or medical care (Hepler and Strand, 1990). Strand et al. categorized eight DRPs as a tool to evaluate pharmacological patient management (Strand et al., 1990) frequently attributed to complex medication regimens correlated with polypharmacy and/or low adherence, as in our study (Table 1).

Multiple studies indicate that patients with diabetes frequently have multiple coexisting chronic conditions like hypertension and coronary artery disease, necessitating a comprehensive approach to treatment, leading to a high risk of polypharmacy. In accordance with that, the most prevalent therapeutic classes among this population in different settings are antidiabetic medications to control blood sugar, lipid-lowering agents manage elevated cholesterol, antiplatelets to prevent blood clots, proton pump inhibitors address gastrointestinal issues, and cardiovascular medications to help manage heart conditions (Alwhaibi et al., 2018; Dobrică et al., 2019; Horii et al., 2019; Larger et al., 2022). The most prevalent medications on DRPs in our study is presented in Table 3 demonstrating the prominence of antidiabetic medications, i.e., insulin and metformin, lipid-lowering agents like statins, antiplatelet drugs such as aspirin, proton pump inhibitors, and cardiovascular medications like clopidogrel and hydrochlorothiazide. The frequent DRPs and recommendations associated with these medications arise from factors like multiple coexisting conditions, complex dosing regimens, side effects, and patient non-adherence. Additional challenges come from therapeutic duplication, physiological changes affecting drug metabolism, and inadequate monitoring, making these classes of drugs particularly susceptible to DRPs. Previous and updated published studies presented compatible data regarding medication class prevalence for DRPs and pharmacist’s recommendations (Snyder et al., 2020; Sheleme et al., 2021).

To better identify integration trends, we categorized DRPs into three groups. The safety-related recommendations in our study had a high acceptance rate (66.8%), compared to both efficacy (55.7%) and informative (48.8%) related categories (Figure 1). Likewise, recommendations with significant and serious severity were also more readily accepted (Figure 2), indicating the non-controversial nature of safety-related DRPs, where compromising patient safety is not an option.

The most frequently documented category of DRPs was the informative category (Figure 1) though with a low acceptance rate (15.7% out of 32.2%). The acceptance rate in this category remained unchanged likely due to the unit’s lack of awareness or a lack of information (e.g., blood tests, experts’ assessments or clarifications) required for optimal pharmacologic patient assessment. Additionally, while the medical team primarily focuses on hospital admission, the pharmacist advocates for a comprehensive overall medication management approach, offering a different perspective on the patient’s current admission to hospitalization. The variance among the clinical focus and goals of the medical team and the clinical pharmacist can occasionally lead to unaccepted recommendations. Therefore, information-related DRP recommendations may not appear essential at first and might be perceived as unproductive before a pharmacist is successfully integrated into the team and prior to the establishment of a robust sense of trust (Byrne et al., 2022).

The pharmacist’s positive impact in our study aligns with prior studies in similar settings, which advocate acceptance and implementation of the pharmacists’ recommendations by medical staff as the most important step in improving patients’ drug therapy. The acceptance rate of recommendations can vary from 11.4% to 94.2% across different study settings and methods (Delpeuch et al., 2015; Alhossan et al., 2016; Stuhec et al., 2019; Al-Taie et al., 2020; Alsuwayni and Alhossan, 2020; Seiberth et al., 2021; Byrne et al., 2022; Rebolledo et al., 2022) and just over 60% in diabetes patients (DeName et al., 2008; Wong, 2017) as demonstrated in the present study.

In our study, the overall rate of acceptance of recommendations increased, reaching 67.6% by the end of the period (Figure 3) with safety and efficacy-related DRP recommendations steadily improving and impressively reaching between 67.6% and 79.4% respectively. This indicates growing reliability and collaboration between pharmacists and healthcare providers over time, enabling expansion into more complex areas as trust improves, as previously described (Teichman and Wan, 2021). This is reflected by the confidence initially built through acceptance of safety-related recommendations, and gradually expanding to the efficacy and informative related recommendations (Figure 3).

As clinical pharmacists are increasingly integrated in different healthcare teams, there’s a growing interest in demonstrating the value of pharmacists in various healthcare settings. The CLEO tool is a multidimensional scale for assessing the potential impact of the pharmacist’s intervention developed by Vo HT et al., in 2019 (V et al., 2021). By using the CLEO tool organizational and economic impacts can be quantified in daily practice of medication review. Tools like CLEO are essential in today’s healthcare environment, where there’s a significant push for value-based care and demonstrating the benefits of interventions beyond clinical outcomes, and therefore was incorporated in different studies with compatible objectives (Durand et al., 2022). Nevertheless, the scope of our study was the evolving integration of the clinical pharmacist into a new unit, as gauged by adherence to the pharmacist’s suggestions. The emphasis on the medical team’s adherence in adopting new medication reviews and integrating recommendations stands apart from the task of measuring the broader organizational and economic consequences of each intervention.

Regarding the diabetic foot population, it has also been demonstrated that when an interdisciplinary team is trained and works together, respect and trust are built and clinical outcomes, such as wound healing, improve as a result (Ogrin et al., 2015). Teams with experience in integrating other healthcare professionals or which had prior relationships with pharmacists find collaboration easier and experience a wide range of acceptance rates (Said et al., 2022). Therefore, integration of the clinical pharmacist is an important step towards achieving such synergy and towards improved pharmacologic outcomes (Singh et al., 2005; Jameson and Baty, 2010; Ip et al., 2013; Game et al., 2016; Rebolledo et al., 2022). Nevertheless, to date, clinical pharmacists are still not fully integrated into most clinical wards, highlighting the importance of addressing this gap.

In order to accomplish successful integration, different evidence-based recommendations have been provided by different guidelines to be applied during the process of integration (Jorgenson et al., 2013; Teichman and Wan, 2021; Said et al., 2022). Better implementation of the following ten recommendations in our study might have resulted in greater integration, as measured by a higher acceptance rate.

• Determine the needs and priorities of the team and its patients
• Develop a pharmacist job description
• Educate the team about the pharmacist’s role
• Pharmacists should educate themselves about other team members’ roles
• Ensure clinic infrastructure supports the pharmacist’s role
• Be highly visible and accessible to the team
• Ensure the pharmacist’s skills are strong and up to date
• Provide proactive care and take responsibility for patient outcomes
• Regularly seek feedback from the team
• Develop and maintain professional relationships with other team members

By understanding the team and its patients, clearly defining roles and responsibilities, developing inter-professional relationships, taking responsibility for patient outcomes and continuously learning and improving professional skills, pharmacists can make a significant impact and become an invaluable member of the primary care team. This has already been implemented in some counties in different extent and variations and therefore is still dynamic (Abousheishaa et al., 2020; Ahmer Raza et al., 2022).

Collaborative efforts improved communication over our 9-month study period, enabling the pharmacist to optimize medical management. This evolving process could be accelerated by implementing strategies such as healthcare provider education and motivation. It should be noted that by the end of the period, the healthcare team found it challenging without the clinical pharmacist’s input and requested permanent clinical pharmacy assistance.

This study has some limitations to be taken into consideration. It was conducted at a single center with a small number of participants with narrow demographic characteristics–mainly, elderly with type two diabetes. However, the small number of participants screened to the study can be attributed to the lack of experience and low motivation to collaborate with the recently implemented pharmacist, although the pace of participants screening improved throughout the study period. Acknowledging regional variations in pharmacist roles, regulations, and educational qualifications in different counties and health systems, a potential limitation of our study is generalizability of our study. In order to avoid generalizability this was addresses and emphasized throughout the study. Despite the potential contextual differences, implementing the recommendations listed and discussed in health systems with advanced regulatory frameworks for pharmacist practice can achieve a successful faster integration. It should further be mentioned, that our work lacks methodological aspects and explanations of how the improvement in acceptance level was achieved, as required by implementation sciences. This lack of linkage between the proposed recommendations and the study outcomes poses a limitation of the full impact and effectiveness of the implementation strategies.

## Conclusion

This study provides a glimpse into the process of the successful integration of a clinical pharmacist in a unit which had not previously experienced the related benefits. The unit’s compliance with the newly integrated pharmacist after a 9-month period and the significantly improved acceptance rate reflect the evolving process of integration to be applicable in different extent in different countries and healthcare systems according to the regulatory frameworks for pharmacist practice. Despite the challenges inherent in the integration process of a clinical pharmacist, barriers can be minimized or prevented by utilizing the suggested strategies.

## Data Availability

The raw data supporting the conclusion of this article will be made available by the authors, without undue reservation.
